# Supplementary material for: Lanostane Triterpenoids and Ergostane Steroids from Ganoderma luteomarginatum and Their Cytotoxicity
Source: Molecules. 2022 Oct 18;27(20):6989. doi: 10.3390/molecules27206989 (PMC9611895; doi:10.3390/molecules27206989)
Supplement: Supplementary file 1 [file molecules-27-06989-s001.zip › molecules-1949877-supplementary.pdf]

Supporting Information

Lanostane Triterpenoids and Ergostane Steroids from *Ganoderma luteomarginatum* and Their Cytotoxicity

Qingyun Ma [1,†], Shuangshuang Zhang [2,†], Li Yang [1], Qingyi Xie [1], Haofu Dai [1], Zhifang Yu [3,*] and Youxing Zhao [1,*]

[1] Haikou Key Laboratory for Research and Utilization of Tropical Natural Products, Institute of Tropical Bioscience and Biotechnology, Chinese Academy of Tropical Agricultural Sciences & Hainan Key Laboratory for Protection and Utilization of Tropical Bioresources, Hainan Academy of Tropical Agricultural Resource, Haikou 571101, China
[2] Jiangsu Food&Pharmaceutical Science College, Huaian 223003, China
[3] College of Food Science and Technology, Nanjing Agricultural University, Nanjing 210095, China
* Correspondence: yuzhifang@njau.edu.cn (Z.Y.); zhaoyouxing@itbb.org.cn (Y.Z.)
† These authors contributed equally to this work.

Figure S1-1 ^{1}H NMR spectrum of compound **1**

Figure S1-2 ^{13}C NMR spectrum of compound **1**

Figure S1-3 HSQC spectrum of compound **1**

Figure S1-4 ^{1}H-^{1}H COSY spectrum of compound **1**

Figure S1-5 HMBC spectrum of compound **1**

Figure S1-6 ROESY spectrum of compound **1**

Figure S1-7 HRESIMS Data of compound **1**

Figure S2-1 ^{1}H NMR spectrum of compound **2**

Figure S2-2 ^{13}C NMR spectrum of compound **2**

Figure S2-3 HSQC spectrum of compound **2**

Figure S2-4 ^{1}H-^{1}H COSY spectrum of compound **2**

Figure S2-5 HMBC spectrum of compound **2**

Figure S2-6 ROESY spectrum of compound **2**

Figure S2-7 HRESIMS Data of compound **2**

Figure S3-1 ^{1}H NMR spectrum of compound **3**

Figure S3-2 ^{13}C NMR spectrum of compound **3**

Figure S3-3 HSQC spectrum of compound **3**

Figure S3-4 ^{1}H-^{1}H COSY spectrum of compound **3**

Figure S3-5 HMBC spectrum of compound **3**

Figure S3-6 ROESY spectrum of compound **3**

Figure S3-7 HRESIMS Data of compound **3**

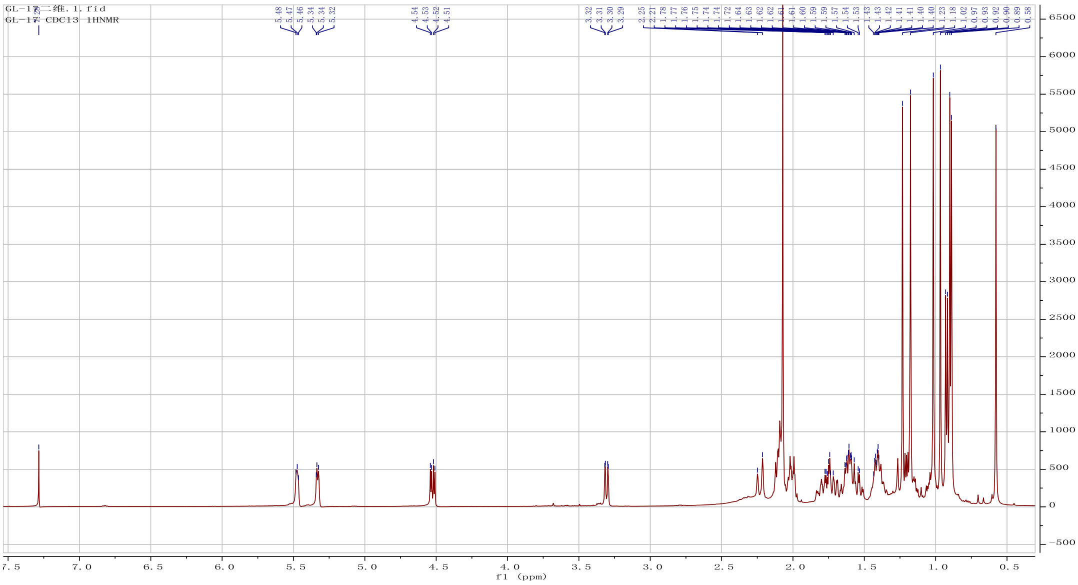

Figure S1-1 ^{1}H NMR spectrum of compound **1**

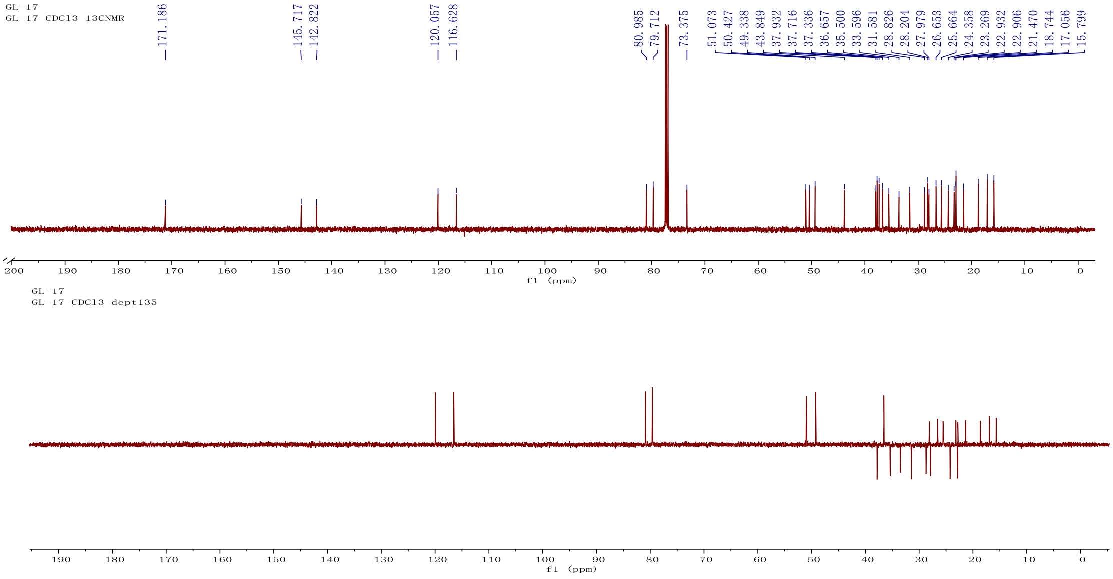

Figure S1-2 ^{13}C NMR spectrum of compound **1**

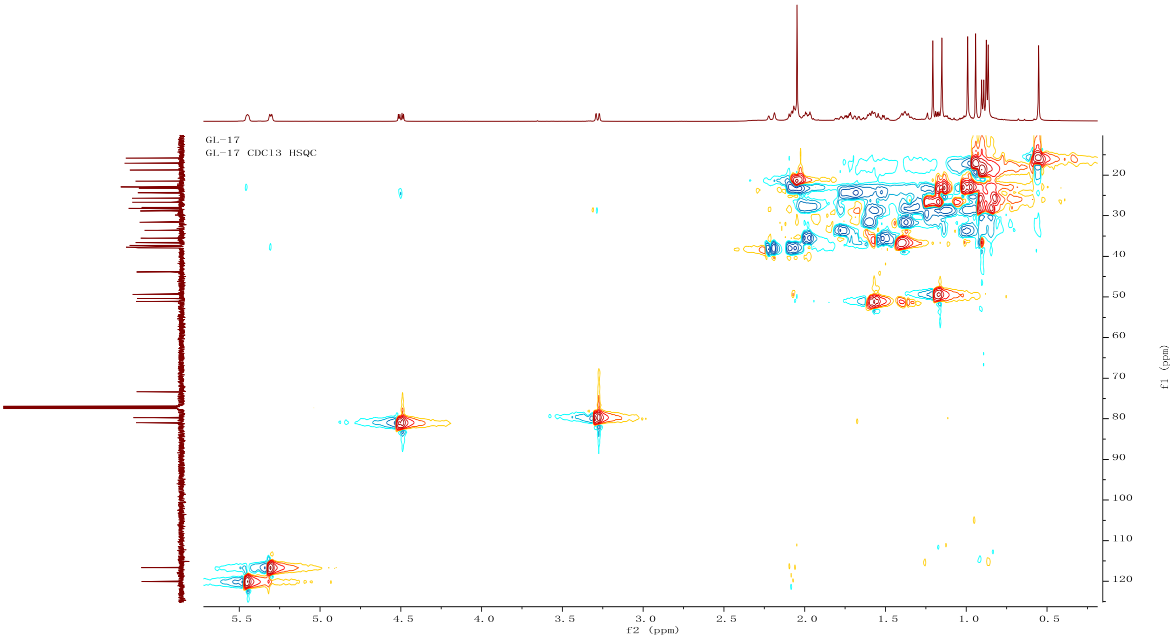

Figure S1-3 HSQC spectrum of compound **1**

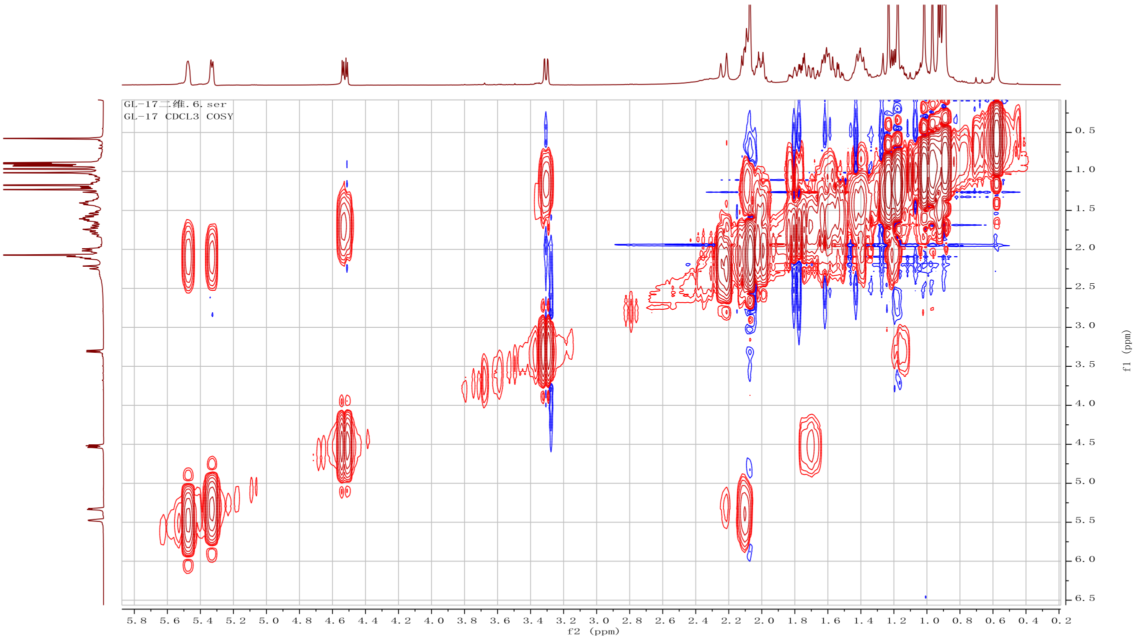

Figure S1-4 ^{1}H-^{1}H COSY spectrum of compound **1**

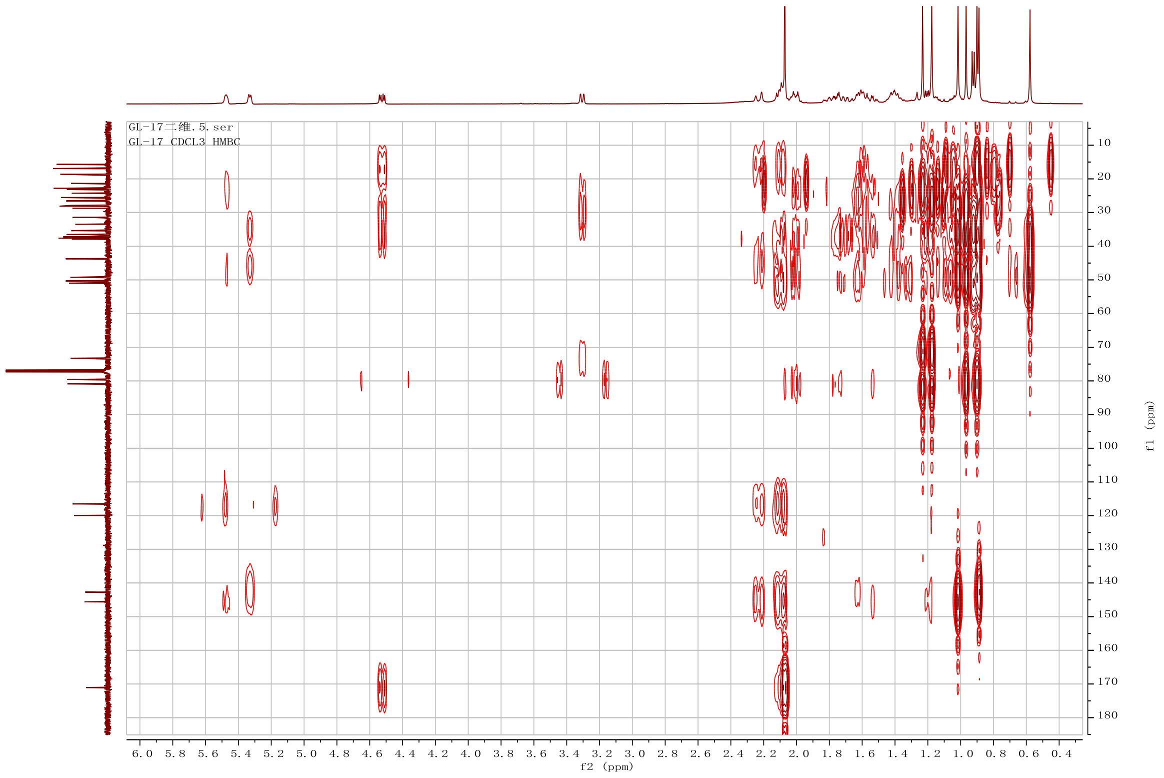

Figure S1-5 HMBC spectrum of compound **1**

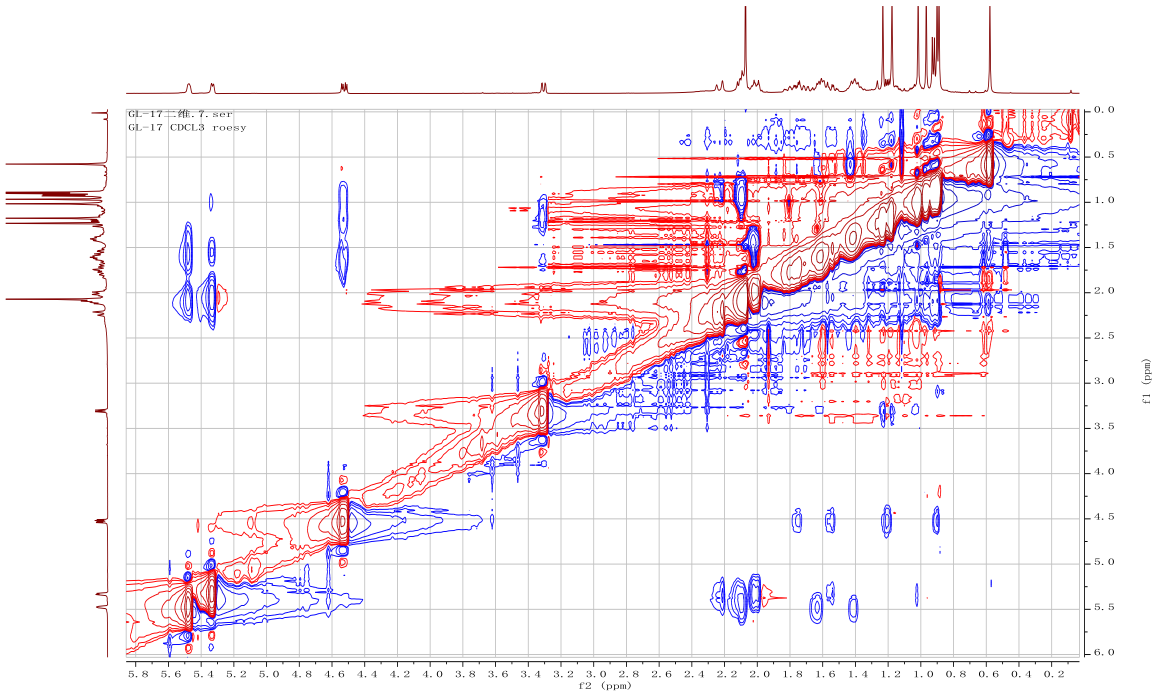

Figure S1-6 ROESY spectrum of compound **1**

Qualitative Analysis Report

**Data Filename**	150325ESIA3.d	**Sample Name**	GL-17
**Sample Type**	Sample	**Position**	
**Instrument Name**	Agilent G6230 TOF MS	**User Name**	KIB
**Acq Method**	ESI.m	**Acquired Time**	3/25/2015 10:54:28 AM
**IRM Calibration Status**	Success	**DA Method**	ESI.m
**Comment**			

**Sample Group**		**Info.**
**Acquisition SW Version**	6200 series TOF/6500 series Q-TOF B.05.01 (B5125.2)	

User Spectra

Fragmentor Voltage	Collision Energy	Ionization Mode
200	0	ESI

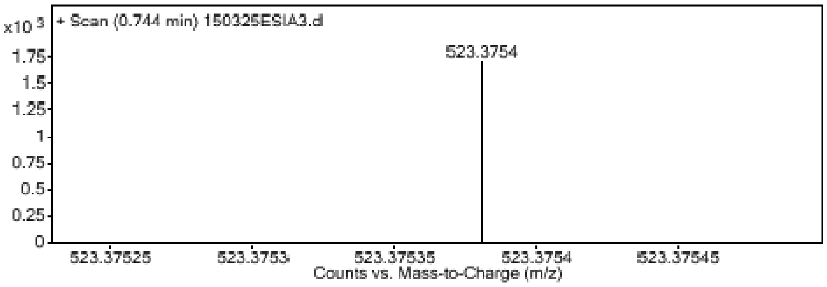

Peak List

m/z	z	Abund
121.0509	1	191469.42
122.0528	1	12813.9
262.1798	1	4608.68
293.1737	1	3099.72
922.0098	1	315840.16
923.0118	1	51887.3
924.0131	1	6379.29
1821.9488	1	18045.1
1822.9516	1	6501.32
1941.9922	1	4096.13

Formula Calculator Element Limits

Element	Min	Max
C	0	200
H	0	400
O	0	12
Na	1	1

Formula Calculator Results

Formula	CalculatedMass	CalculatedMz	Mz	Diff. (mDa)	Diff. (ppm)	DBE
C32 H52 Na O4	523.3763	523.3758	523.3754	1.7	3.3	6.5000

--- End Of Report ---

Figure S1-7 HRESIMS Data of compound **1**

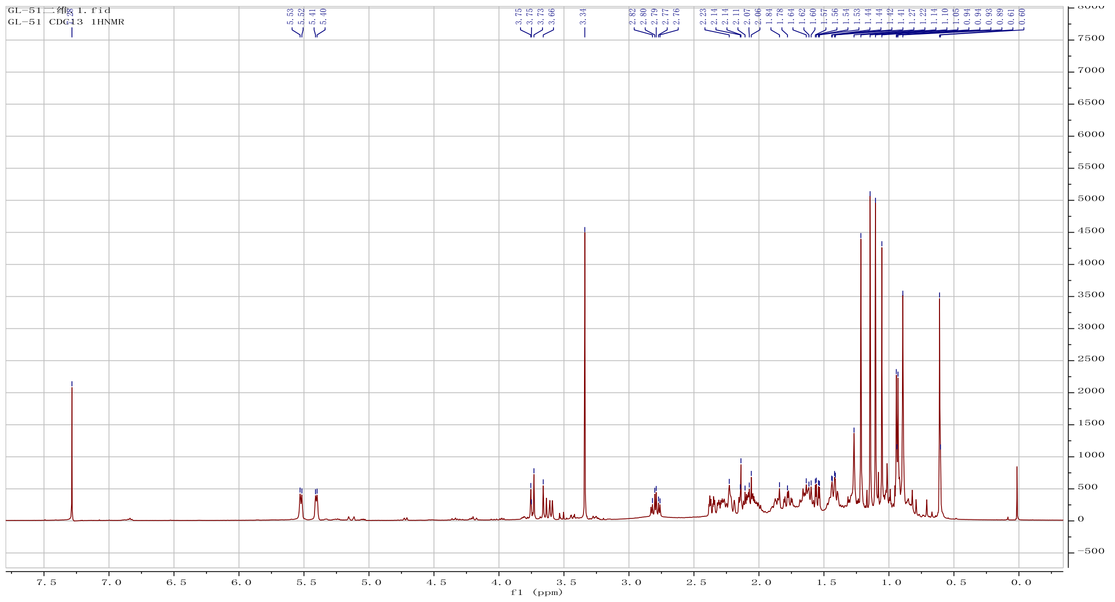

Figure S2-1 ^{1}H NMR spectrum of compound **2**

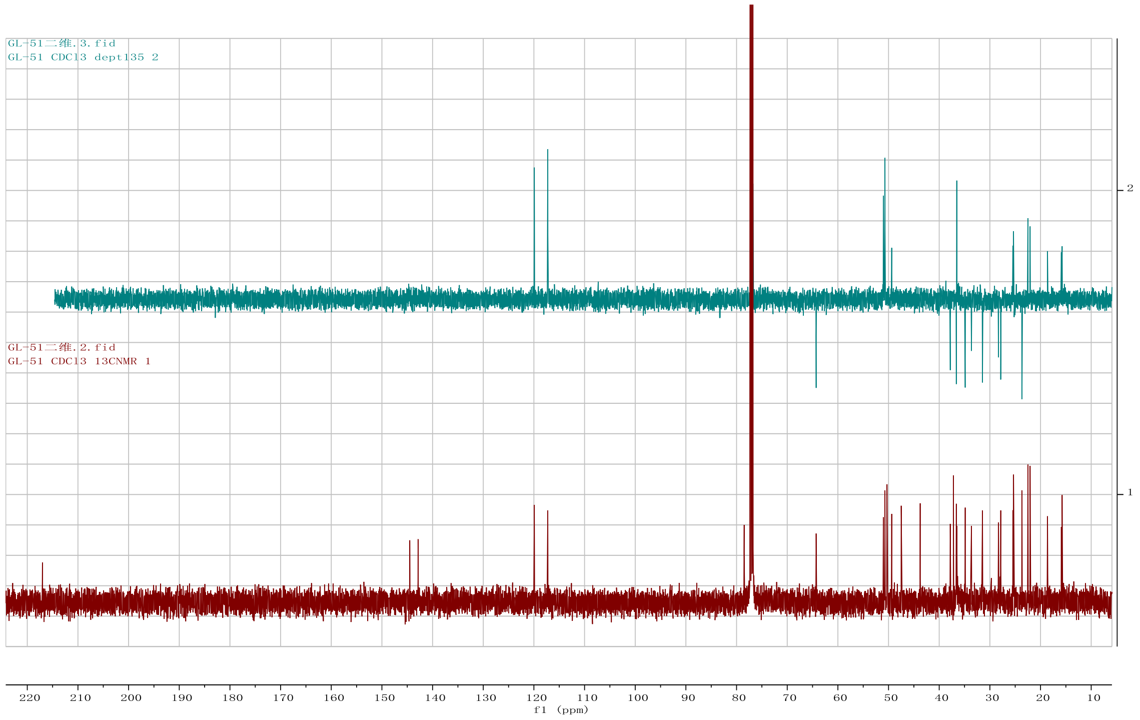

Figure S2-2 ^{13}C NMR spectrum of compound **2**

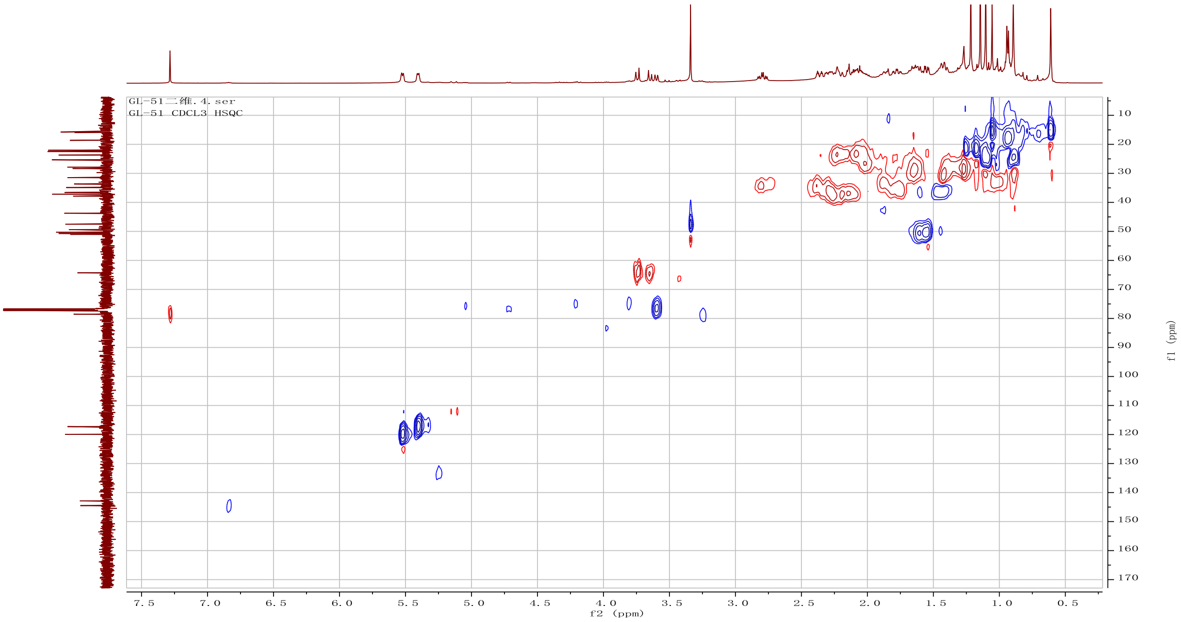

Figure S2-3 HSQC spectrum of compound **2**

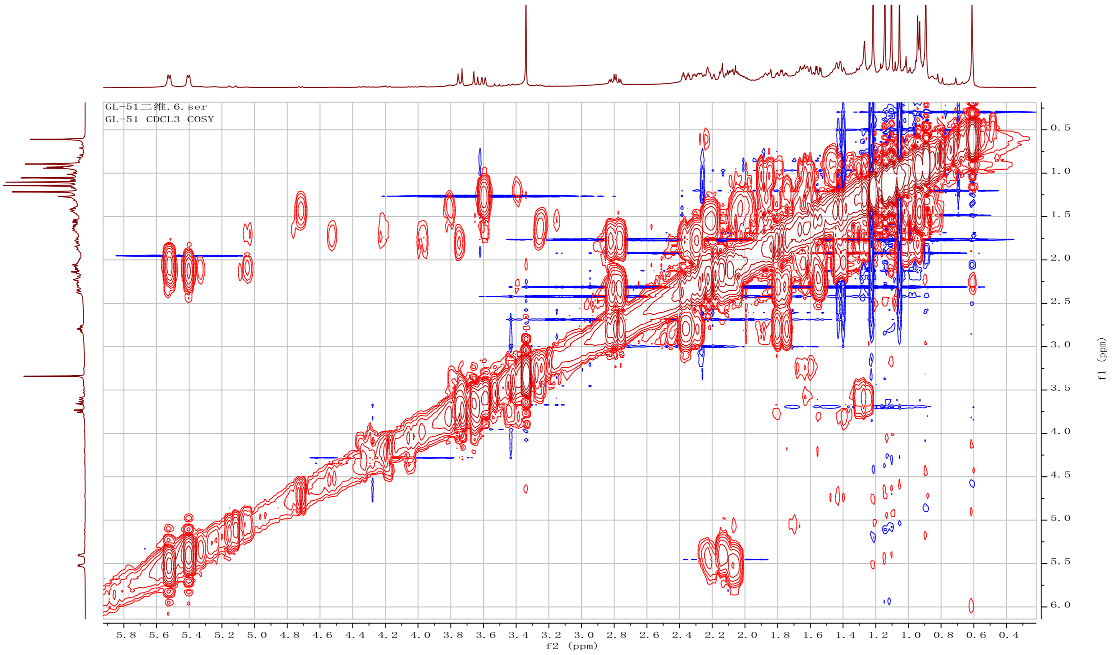

Figure S2-4 ^{1}H-^{1}H COSY spectrum of compound **2**

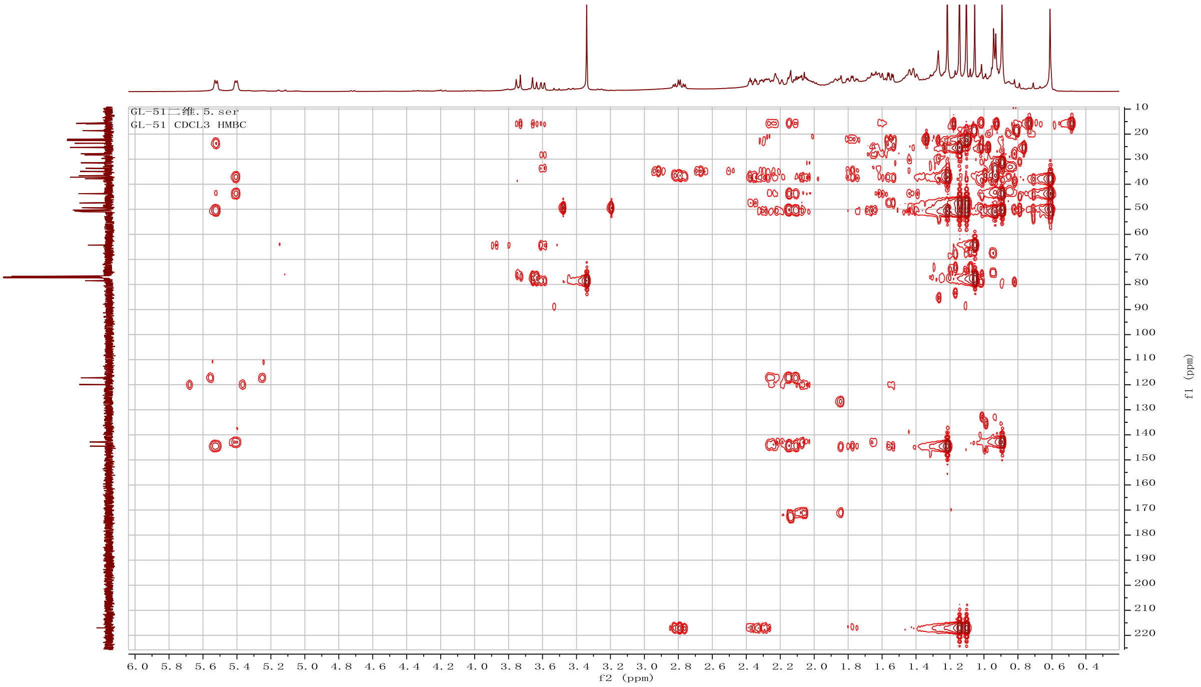

Figure S2-5 HMBC spectrum of compound **2**

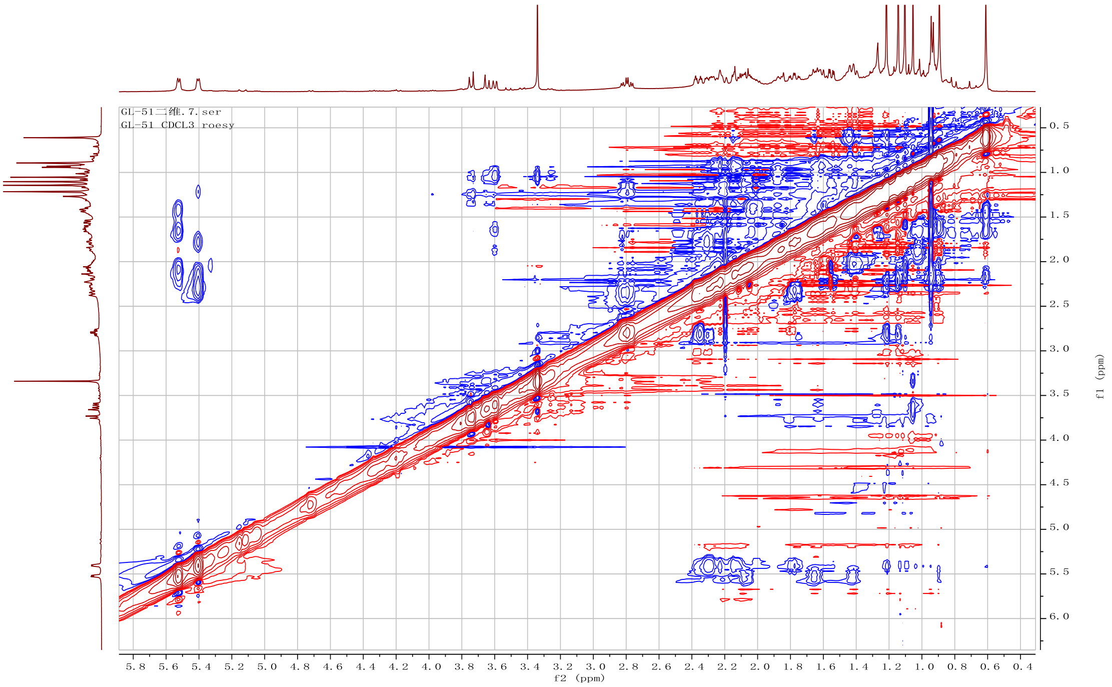

Figure S2-6 ROESY spectrum of compound **2**

Qualitative Analysis Report

**Data Filename**	150107ESIA7.d	**Sample Name**	GL-51
**Sample Type**	Sample	**Position**	
**Instrument Name**	Agilent G6230 TOF MS	**User Name**	KIB
**Acq Method**	ESI.m	**Acquired Time**	1/7/2015 8:45:29 AM
**IRM Calibration Status**	[redacted]	**DA Method**	ESI.m
**Comment**			

Sample Group **Info.**
Acquisition SW Version 6200 series TOF/6500 series Q-TOF B.05.01 (B5125.2)

User Spectra

Fragmentor Voltage	Collision Energy	Ionization Mode
200	0	ESI

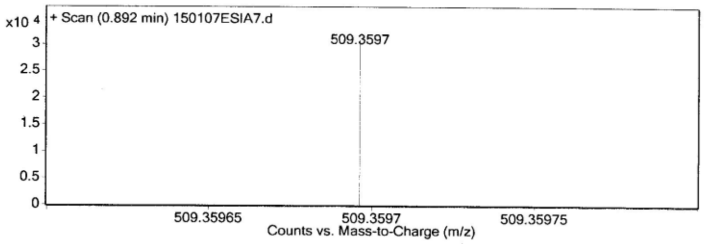

Peak List

m/z	z	Abund	Formula	Ion
121.0509	1	48950.69		
481.2925	1	11706.12		
509.3597	1	30718.59	C31 H50 Na O4	M+
510.3619	1	10323.14	C31 H50 Na O4	M+
922.0098	1	85934.12		
923.0115	1	14322.79		
995.7264	1	21669.21		
996.7311	1	13986.23		
1023.7229	1	18231.22		
1024.7269	1	11303.9		

Formula Calculator Element Limits

Element	Min	Max
C	0	200
H	0	400
O	0	9
Na	1	1

Formula Calculator Results

Formula	CalculatedMass	Mz	Diff.(mDa)	Diff. (ppm)	DBE
C31 H50 Na O4	509.3607	509.3597	1.0	1.9	6.5

--- End Of Report ---

Figure S2-7 HRESIMS Data of compound **2**

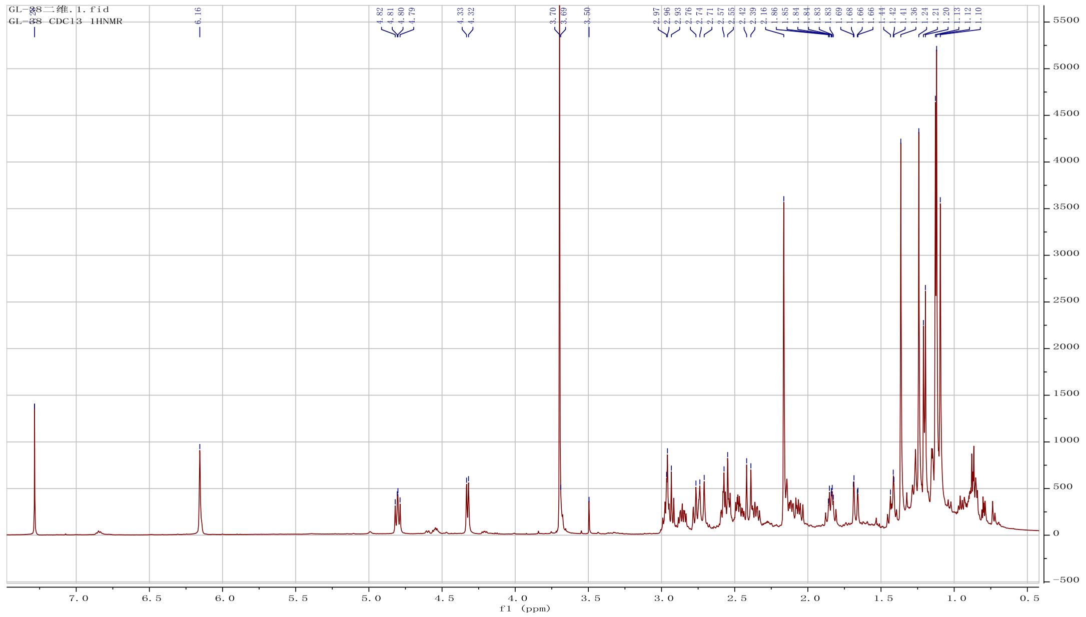

Figure S3-1 ^{1}H NMR spectrum of compound **3**

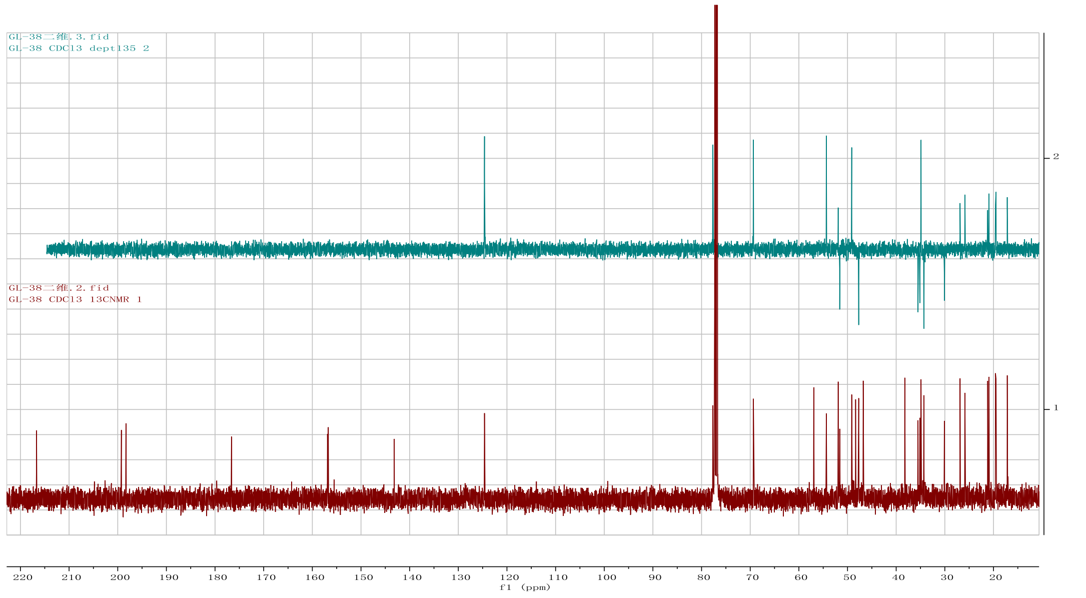

Figure S3-2 ^{13}C NMR spectrum of compound **3**

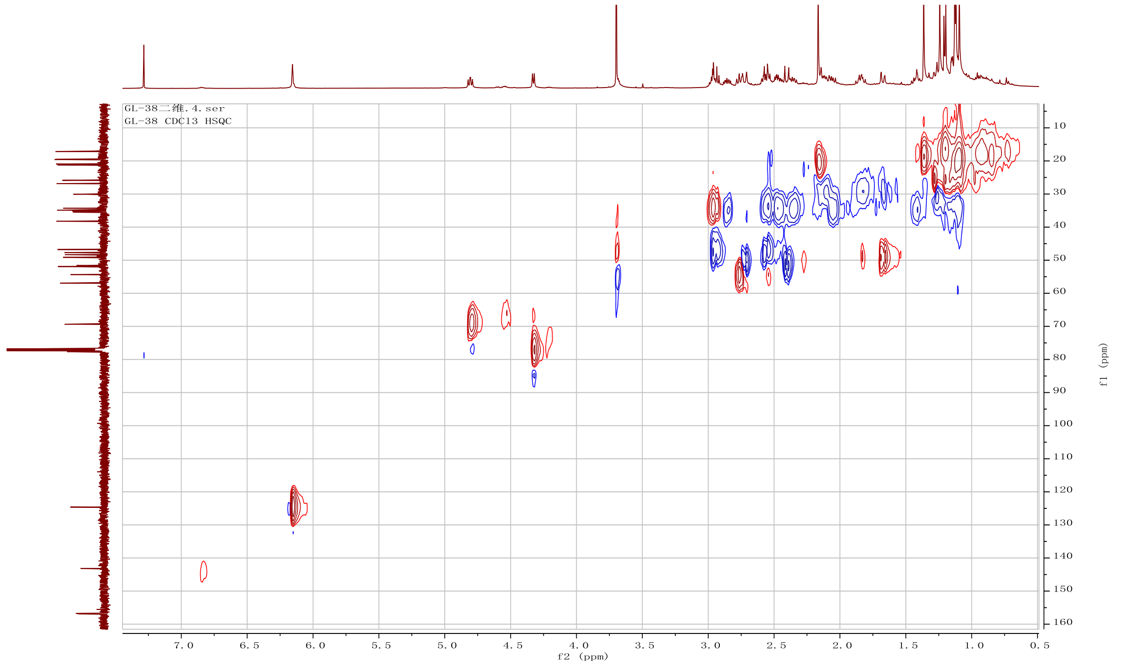

Figure S3-3 HSQC spectrum of compound **3**

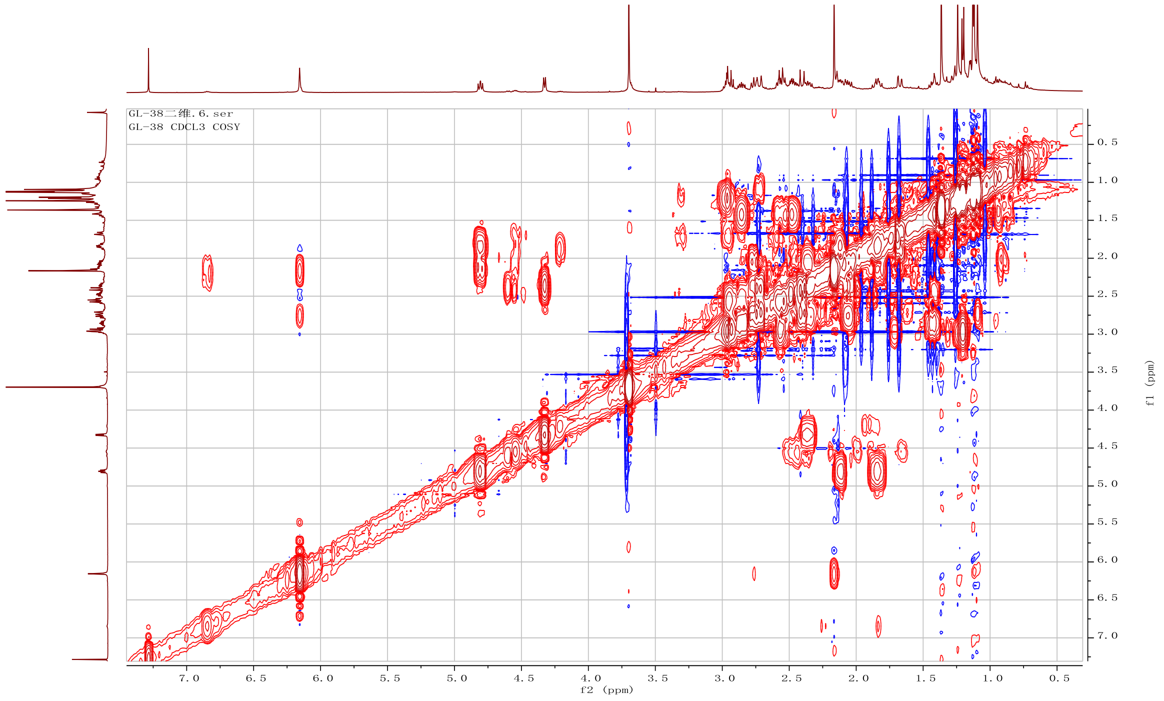

Figure S3-4 ^{1}H-^{1}H COSY spectrum of compound **3**

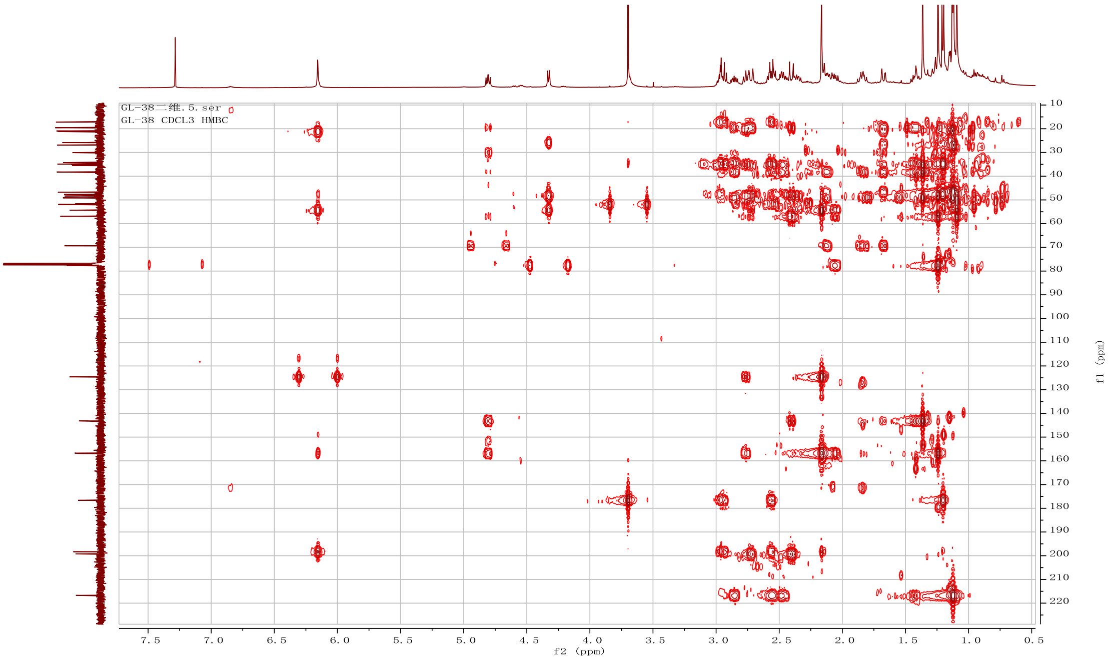

Figure S3-5 HMBC spectrum of compound **3**

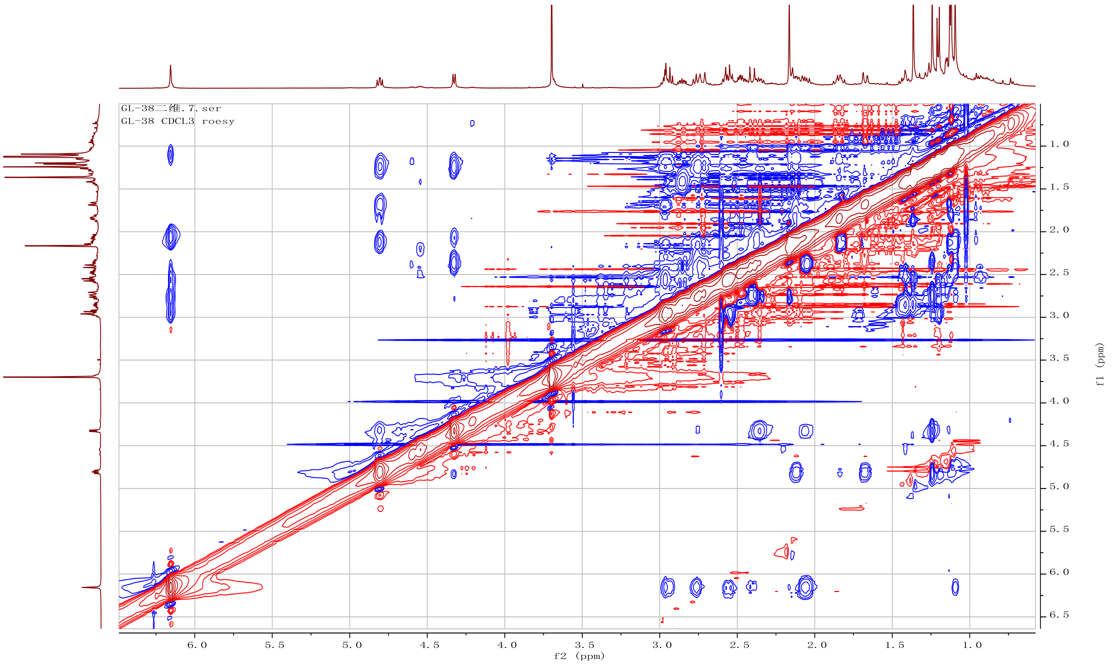

Figure S3-6 ROESY spectrum of compound **3**

Qualitative Analysis Report

**Data Filename**	150107ESIA6.d	**Sample Name**	GL-38
**Sample Type**	Sample	**Position**	
**Instrument Name**	Agilent G6230 TOF MS	**User Name**	KIB
**Acq Method**	ESI.m	**Acquired Time**	1/7/2015 8:43:52 AM
**IRM Calibration Status**	Success	**DA Method**	ESI.m
**Comment**			

**Sample Group**		**Info.**
**Acquisition SW Version**	6200 series TOF/6500 series Q-TOF B.05.01 (B5125.2)	

User Spectra

Fragmentor Voltage	Collision Energy	Ionization Mode
200	0	ESI

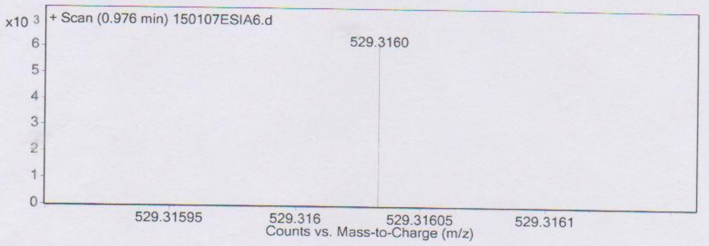

Peak List

m/z	z	Abund
121.0509	1	61042.15
182.0393		8911.58
215.0284		8792.27
232.1116		9777.82
284.3306	1	12744.35
312.3621		8034.82
383.1958	1	10188.46
551.3002	1	11822.61
922.0098	1	86749.96
923.0124	1	13980.35

Formula Calculator Element Limits

Element	Min	Max
C	0	200
H	0	400
O	0	9

Formula Calculator Results

Formula	CalculatedMass	Mz	Diff.(mDa)	Diff. (ppm)	DBE
C31 H45 O7	529.3165	529.3160	0.5	1.0	9.5

--- End Of Report ---

Figure S3-7 HRESIMS Data of compound **3**
